# A Comprehensive Analysis of Diagnostic and Virological Surveillance During the 2023–2025 Measles Epidemic Scenario

**DOI:** 10.3390/diagnostics16071109

**Published:** 2026-04-07

**Authors:** Martina Franceschiello, Martina Tamburello, Giulia Piccirilli, Eva Caterina Borgatti, Federica Lanna, Alessia Bertoldi, Simona Venturoli, Giada Rossini, Silvia Gioacchini, Melissa Baggieri, Fabio Magurano, Michela Morri, Giulio Matteo, Christian Cintori, Giovanna Mattei, Vittorio Lodi, Liliana Gabrielli, Tiziana Lazzarotto

**Affiliations:** 1 Microbiology, Department of Medical and Surgical Sciences, University of Bologna, 40138 Bologna, Italy; marti.franceschiello@studio.unibo.it (M.F.); martina.tamburello2@unibo.it (M.T.); evacaterina.borgatti@studio.unibo.it (E.C.B.); federica.lanna4@studio.unibo.it (F.L.); tiziana.lazzarotto@unibo.it (T.L.); 2 Microbiology Unit, IRCCS Azienda Ospedaliero-Universitaria di Bologna, 40138 Bologna, Italy; alessia.bertoldi3@unibo.it (A.B.); simona.venturoli@aosp.bo.it (S.V.); giada.rossini@aosp.bo.it (G.R.); liliana.gabrielli@aosp.bo.it (L.G.); 3 Department of Infectious Diseases, Istituto Superiore di Sanità, 00161 Rome, Italy; silvia.gioacchini@iss.it (S.G.); melissa.baggieri@iss.it (M.B.); fabio.magurano@iss.it (F.M.); 4 Unit of Hygiene and Public Health Rimini, Department of Public Health, Romagna Local Health Authority, 47624 Rimini, Italy; michela.morri@auslromagna.it; 5 Settore Prevenzione Collettiva e Sanità Pubblica—Direzione Generale Cura della Persona, Salute e Welfare, Emilia-Romagna Region, 40127 Bologna, Italy; giulio.matteo@regione.emilia-romagna.it (G.M.); christian.cintori@regione.emilia-romagna.it (C.C.); giovanna.mattei@regione.emilia-romagna.it (G.M.); 6 SSD Sorveglianza Sanitaria, IRCCS Azienda Ospedaliero-Universitaria di Bologna, 40138 Bologna, Italy; vittorio.lodi@aosp.bo.it

**Keywords:** measles virus, laboratory diagnosis, molecular characterisation, surveillance activities, differential diagnosis

## Abstract

**Background/Objectives**: Since 2023, a significant increase in measles cases has been reported worldwide, and Italy has been among the most affected European countries. In this context, the integration of laboratory and epidemiological data enables timely case classification and helps distinguish between imported and indigenous cases, supporting disease control. However, most studies address only selected aspects of surveillance. Therefore, this study aimed to provide an integrated analysis of virological and epidemiological surveillance activities conducted between November 2023 and December 2025 by the Regional Reference Laboratory in the Emilia-Romagna Region (ERR). **Methods**: A total of 806 clinical samples (269 urine, 267 oral fluids—saliva or oropharyngeal swabs—and 270 sera) from 291 suspected measles cases were tested by molecular and/or serological methods, and MV genotyping was performed. Samples from discarded cases were also analysed for parvovirus B19 (B19V), human herpesvirus 6 (HHV-6), enterovirus (EV), and varicella zoster virus (VZV), chikungunya virus (CHIKV) and dengue virus (DENV). **Results**: Of 291 suspected cases, 176 (60.5%) were confirmed. Median age was 33 years, with 46% in the 15–39 year group. Vaccination status was available for 165: 90.3% were unvaccinated, 5.4% had one dose, and 4.2% had two doses. Notably, over half of confirmed cases occurred in areas with vaccine-hesitant communities. MV strain characterisation was performed in 99.4% of MV-RNA positive cases, with 84.3% genotype D8 and 15.6% genotype B3; 83% of strains were of indigenous origin, suggesting an ongoing endemic circulation. Clinical data showed complications in 19.3%, mainly pneumonia and diarrhoea. Additionally, differential diagnosis enabled the identification of the etiological agent in 37.5% of measles/rubella discarded cases, and 37.6% (29/77) tested positive for B19V. **Conclusions**: The study results highlight that effective measles surveillance must be supported by integrating timely virological diagnosis, molecular and epidemiological investigations, and differential diagnosis, to achieve the WHO goals of eliminating measles transmission.

## 1. Introduction

Measles is a highly contagious disease caused by the measles virus (MV), a single-stranded RNA virus of the family Paramyxoviridae, genus Morbillivirus. It is typically characterised by fever, cough, coryza, conjunctivitis, and a maculopapular rash that usually begins on the face and spreads caudally; small white lesions on the buccal mucosa (Koplik’s spots), representing the characteristic enanthem of measles, may appear two to three days after symptom onset [1]. However, in individuals with pre-existing immunity or in immunocompromised patients, clinical presentation may be modified, with mild, absent or atypical rash, making diagnosis more challenging [2,3]. Measles may also lead to severe complications, including pneumonia, diarrhoea, encephalitis, and death [4].

To eliminate measles, the World Health Organisation (WHO) plans to ensure high vaccination coverage and to implement effective surveillance systems, as outlined in the WHO European Vaccine Action Plan 2015–2020 and in the European Immunization Agenda 2030 (EIA 2030) [5]. Despite the initiative, since 2023, a concerning re-emergence of measles has been reported, with a worldwide increase in measles cases [6]. The WHO European Region also documented an alarming rise, with over 30,000 cases in 40 of the 53 countries, including large outbreaks in Romania, Austria, and France [7,8,9]. Italy also experienced a significant measles resurgence, with 1,045 cases in 2024, and continuously rising numbers in 2025. In the north-east of Italy, the Emilia-Romagna Region (ERR) ranked third for reported measles cases, after Lazio and Sicily [10,11,12].

In line with the WHO goals, Italy developed the National Plan for the Elimination of Measles and Congenital Rubella (PNEMoRc), which has been progressively strengthened through the introduction of laboratory confirmation protocols and integrated surveillance systems [13]. A significant advancement was achieved in 2017 with the establishment of the Measles and Rubella Network (MoRoNet). The network consists of 15 accredited subnational laboratories coordinated by the National Reference Laboratory (NRL) at the National Institute of Health (ISS) in Rome [14]. The Laboratory of Virology at the IRCCS Azienda Ospedaliero-Universitaria di Bologna has been designated by the ERR authorities as the Regional Reference Laboratory (LRR). In 2017, it was accredited by NRL as LRR01 [14,15]. Speciments from all the suspected measles cases referred to the Local Health Authorities (LHAs) were sent to LRR01 for laboratory confirmation and MV genotyping [5]. According to the integrated surveillance, laboratory confirmation was carried out by the detection of anti-measles IgM antibodies and/or viral RNA using real-time RT-PCR [16], along with simultaneous testing for rubella virus (RV). Beginning from March 2024, several European countries also reported a substantial increase in the incidence of parvovirus B19 (B19V) [17,18] and its co-circulation with MV [19]. Due to the clinical similarity among these infections, the introduction of additional laboratory methods for differential diagnosis is crucial in order to identify other fever and rash-associated viruses, such as B19V, human herpesvirus 6 (HHV-6), enterovirus (EV), and others [20].

In this setting, the integration of laboratory and epidemiological data enables timely case classification and helps distinguish between imported and indigenous cases, preventing measles spread. This integrated approach is particularly important during periods of measles re-emergence, when rapid case assessment and accurate interpretation of transmission patterns are essential for public health response. However, most studies address only selected aspects of surveillance. Therefore, this study aimed to provide an integrated analysis of surveillance activities conducted between November 2023 and December 2025 by the Regional Reference Laboratory in the ERR. Specifically, the investigation focused on: (i) the crucial role of virological diagnosis and active surveillance in confirming measles cases, (ii) the utility of molecular characterisation to identify circulating MV strains and to support epidemiological investigation in tracing transmission pathways, and (iii) the importance of including other viruses responsible for febrile rash illnesses in the differential diagnosis.

## 2. Materials and Methods

### 2.1. Sample Collection

From November 2023 to December 2025, 291 suspected measles cases were reported in ERR. A total of 806 clinical samples (269 urine, 267 oral fluids—saliva or oropharyngeal swabs—and 270 sera) from 281 patients with suspected measles infection were processed at LRR01 for laboratory diagnosis and genotyping of MV strains. Notably, 10/291 (3.4%) patients declined sample collection for laboratory investigations. A patient information form containing personal and clinical data, including date of rash onset and vaccination status, was provided along with the specimens, in accordance with the PNEMoRc [13]. Case classification (confirmed, probable, or possible) was based on the European Commission case definition [21]. For each case, the ERR Health Authorities provided additional data related to the type of transmission (sporadic, outbreak/chain of transmission-related) and the source of infection (imported, importation-related, or indigenous) [22,23,24]. Suspected cases that tested negative for both MV and RV by molecular and/or serological methods were defined as discarded cases.

### 2.2. Laboratory Methods

#### 2.2.1. Serological Test

Serum specimens were tested for the detection of MV-IgM and IgG using the LIAISON^®^ measles IgM/IgG kit on the LIAISON^®^ XL instrument (DiaSorin, Saluggia, Italy), following the manufacturer’s instructions. Specimens that tested negative for MV-IgM were tested for RV-IgM and IgG using the Elecsys^®^ Rubella IgG/IgM kit on the Cobas^®^ 8000 instrument (Roche Diagnostics, Monza, Italy). Results were provided as quickly as possible, within a median time of 24 h after specimen receipt.

#### 2.2.2. Molecular Test

The simultaneous detection of MV and RV RNA was performed on urine and oral fluid specimens by a homemade multiplex real-time RT-PCR assay, adaptation of the protocol of Hübschen et al. [25]. Specifically, RNA extraction was carried out using the ELITe InGenius^®^ SP 200 extraction kit on the ELITe InGenius^®^ instrument (ELITechGroup, Bruker Corporation, Torino, Italy), according to the manufacturer’s instructions. Five microliters of extracted RNA were amplified by multiplex Real-Time RT-PCR using the TaqPath™ 1-Step Multiplex Master Mix (No ROX) (Applied Biosystems™, Thermofisher Scientific, Waltham, MA, USA) and the set of primers and TaqMan probes targeting the nucleoprotein (N) gene of MV, and the p150 gene of RV [25]. Results were provided as quickly as possible, within a median time of 24 h after specimen receipt.

#### 2.2.3. Sequencing and Phylogenetic Analysis

MV strain characterisation was performed on MV-RNA positive cases by amplifying and sequencing the highly variable 450-nucleotide (nt) region at the C-terminal of the nucleoprotein (N-450), according to WHO guidelines [16]. Ten microliters of extracted RNA were reverse transcribed using the LunaScript^®^ RT SuperMix Kit (New England Biolabs, Inc., Ipswich, MA, USA), followed by semi-nested PCR performed with the Taq PCR Master Mix kit (QIAGEN, Hilden, Germany) and the primer set described by Alla et al. [26]. Sequences obtained by the Sanger method [27] were examined using Chromas version 2.23 (Technelysium, Brisbane, Australia), aligned with Clustal Omega multiple sequence alignment (MSA, online tool available at www.ebi.ac.uk (accessed on 4 November 2025)), and analysed with the Basic Local Alignment Search Tool-nucleotide (BLASTn, online tool available at https://blast.ncbi.nlm.nih.gov/Blast.cgi (accessed on 4 November 2025)) to identify similarities with previously reported strains. Sequences were named according to the official WHO nomenclature [28] and sent to NRL for submission to the WHO MeaNS (Measles Nucleotide Surveillance) database obtaining a Distinct Sequence Identifier (DSId) [29]. Results were provided within 30 days after rash onset. The phylogenetic tree was constructed with MEGA12, using the maximum likelihood method, and the Kimura 2-parameter model of nucleotide substitutions. Reference sequences for genotypes D8 (MVi/Manchester.GBR/30.94) and B3 (MVi/New York.USA/0.94) were used as outgroups.

### 2.3. Differential Diagnosis

Samples from discarded cases were tested for the detection of the main viruses causing rash-associated diseases, such as B19V, HHV-6, EV, and varicella zoster virus (VZV). Although VZV is typically characterised by a vesicular rash, in some cases during the early stages of the disease, the lesions may take on maculo-papular characteristics similar to those of MV [30,31]. For this reason, VZV was included in the differential diagnosis. During the study period, autochthonous outbreaks of chikungunya virus (CHIKV) and dengue virus (DENV) were documented in the ERR [32,33]. To ensure a comprehensive diagnosis, measles/rubella discarded cases reported during these outbreaks, or with a travel history to endemic areas, were also tested for CHIKV and DENV (12 and 6 out of 105, respectively).

Specifically, B19V-DNA was extracted from serum samples using the ELITe InGenius^®^ SP 200 extraction kit and quantified with the Parvovirus B19 ELITe MGB^®^ Kit on the ELITe InGenius^®^ instrument (ELITechGroup, Bruker Corporation, Torino, Italy). Oral fluid samples were tested for the detection of HHV-6, EV, and VZV; serum samples for CHIKV and DENV. Specifically, nucleic acids were extracted using the AltoStar^®^ Purification Kit 1.5 on the AltoStar^®^ Automation System AM16 instrument (Altona Diagnostics, Hamburg, Germany), and viral quantification was performed using three commercial assays, according to the manufacturers’ instructions: RealStar^®^ HHV-6 PCR Kit 1.0, RealStar^®^ Enterovirus RT-PCR Kit 1.0, RealStar^®^ VZV PCR Kit 1.0, RealStar^®^ Chikungunya RT-PCR Kit 2.0, RealStar^®^ Dengue RT-PCR kit 3.0 (Altona Diagnostics, Hamburg, Germany).

## 3. Results

### 3.1. Laboratory Results and Case Classification

Between November 2023 and December 2025, the surveillance system of ERR reported 291 suspected measles cases, collecting samples from 281 patients (96.6%). Of these, 176 tested positive by molecular and/or serological methods. Specifically, 88.1% (155/176) were confirmed by both assays, 6.8% (12/176) by molecular test only, and 5.1% (9/176) by MV-IgM detection alone. Notably, the confirmation with only one of the two methods (13/21) was mainly due to the unavailability of the appropriate sample (Supplementary Table S1).

**Figure 1:**
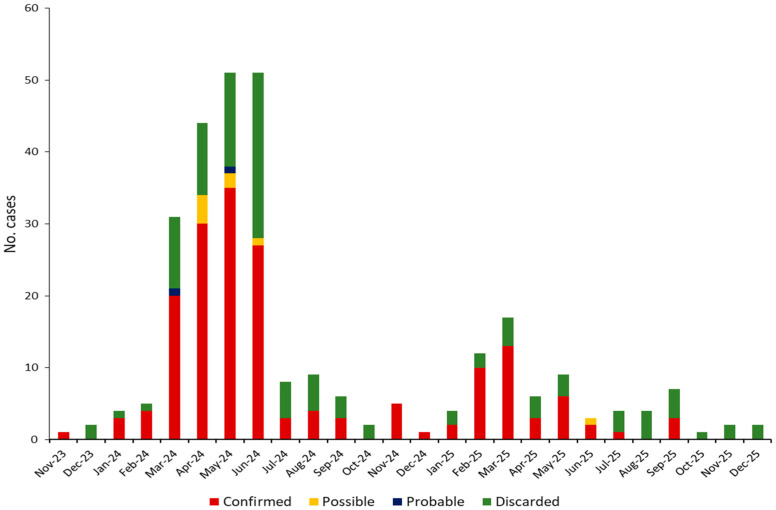
Distribution of measles cases (confirmed, possible, probable, and discarded) notified in ERR by rash onset from November 2023 to December 2025.

Based on laboratory results and epidemiological/clinical data, the 291 suspected cases were classified as follows: 176 (60.5%) confirmed, 8 (2.7%) possible (clinically compatible), 2 (0.7%) probable (epidemiologically linked). The remaining 105 (36.1%) cases, which tested negative for both MV and RV, were classified as discarded. The majority of suspected and confirmed measles cases were notified between March and June 2024, followed by a progressive decline (Figure 1). A slight increase was also observed in the early months of 2025, with a peak in March.

### 3.2. Confirmed Cases

#### 3.2.1. Demographic and Clinical Characteristics

**Figure 2:**
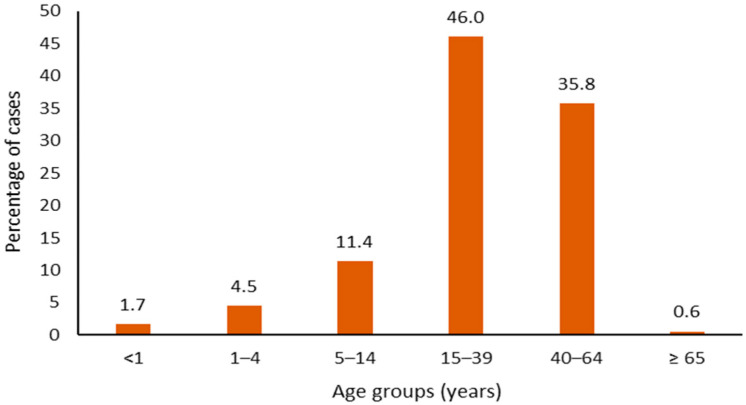
Distribution of confirmed cases (*n* = 176) by age groups.

Among the 176 confirmed cases, 105 were males (59.7%), and 71 were females (40.3%); the median age was 33 years (range: 8 months–67 years). Most of the confirmed cases (*n* = 81, 46%) occurred in the 15–39 age group (Figure 2). Three cases (1.7%) involved individuals in regular contact with patients: two were healthcare workers (HCWs)—defined as any hospital or other healthcare staff—and one was a cultural mediator. None of them was a staff member at the National Health Service (NHS).

**Figure 3:**
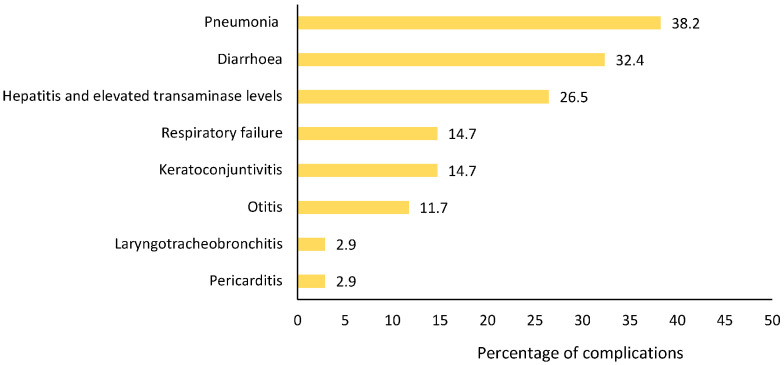
Percentage and type of complications reported for 34 out of 176 confirmed measles cases.

Complications were reported in 34/176 (19.3%) patients, with pneumonia and diarrhoea being the most common (Figure 3); twelve cases experienced multiple complications. All patients were unvaccinated, except for one case, who received two doses in a non-EU country. Overall, 68% (23/34) of patients with complications required hospitalisation.

#### 3.2.2. Vaccination Status

Vaccination status was known for 165 out of 176 cases (93.7%): 149 (90.3%) were unvaccinated, 9 (5.4%) vaccinated with one dose—including one individual who had received a measles-containing vaccine as prophylaxis after being identified as a contact of a confirmed case—and 7 (4.2%) vaccinated with two doses. Of the unvaccinated cases, three were infants under 12 months of age and therefore not eligible for vaccination. Furthermore, 50.6% (89/176) of confirmed cases were reported in an area with a high prevalence of vaccine hesitancy. In this area, vaccination coverage as of December 2024 ranged from 91.9% to 93.9% for the first dose by 24 months of age and from 83.6% to 85.5% for the full vaccination cycle by 7 years of age.

#### 3.2.3. Epidemiological Data and Molecular Characterisation of MV Strains

Epidemiological investigation showed that among the 176 confirmed cases, 83 (47.2%) were associated with 36 distinct outbreaks or chains of transmission. Of these, 28 occurred in 2024, and 8 in 2025. The remaining 93 cases (52.8%) were reported as sporadic (Supplementary Figure S1). Additionally, 146 cases (83%) were referred to as locally acquired (indigenous), whereas, 26 cases (14.7%) had a travel history to countries including Egypt, Ireland, Malaysia, Moldova, Morocco, Pakistan, Romania, Russia, Spain, Turkey, the United Kingdom, and Vietnam, and were classified as imported. Within this group, five cases were classified as outbreak/chain of transmission-related cases, and 21 as sporadic. Furthermore, four (2.3%) were epidemiologically linked to the travel-related cases and therefore classified as importation-related. Notably, one of the 36 outbreaks involved passengers on a flight from North Africa to Bologna and was reported in 2025, during the epidemiological week 5.

**Figure 4:**
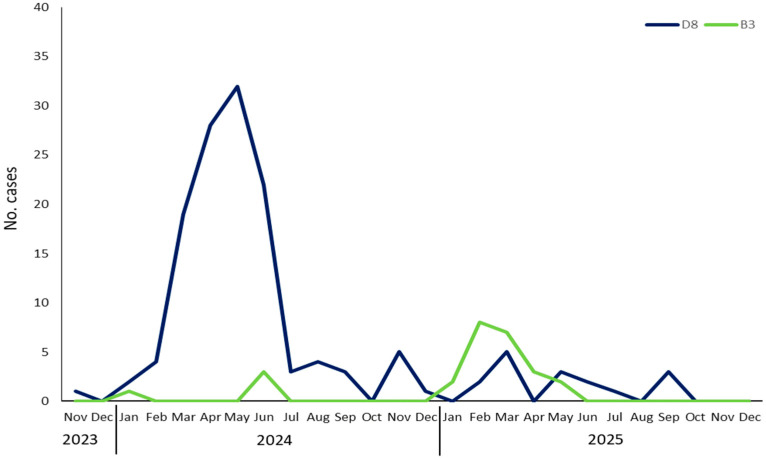
Genotype circulation in ERR, from November 2023 to December 2025.

Molecular characterisation was performed in 166/167 MV-RNA positive cases (99.4%), identifying two distinct genotypes: D8 and B3. In one case, genotyping was not feasible due to a low MV-RNA load. Genotype D8 was the most prevalent (140/166, 84.3%) and primarily associated with the 2024 epidemic, whereas genotype B3 was identified in 15.6% (26/166) of cases and predominantly detected during 2025 (Figure 4).

**Figure 5:**
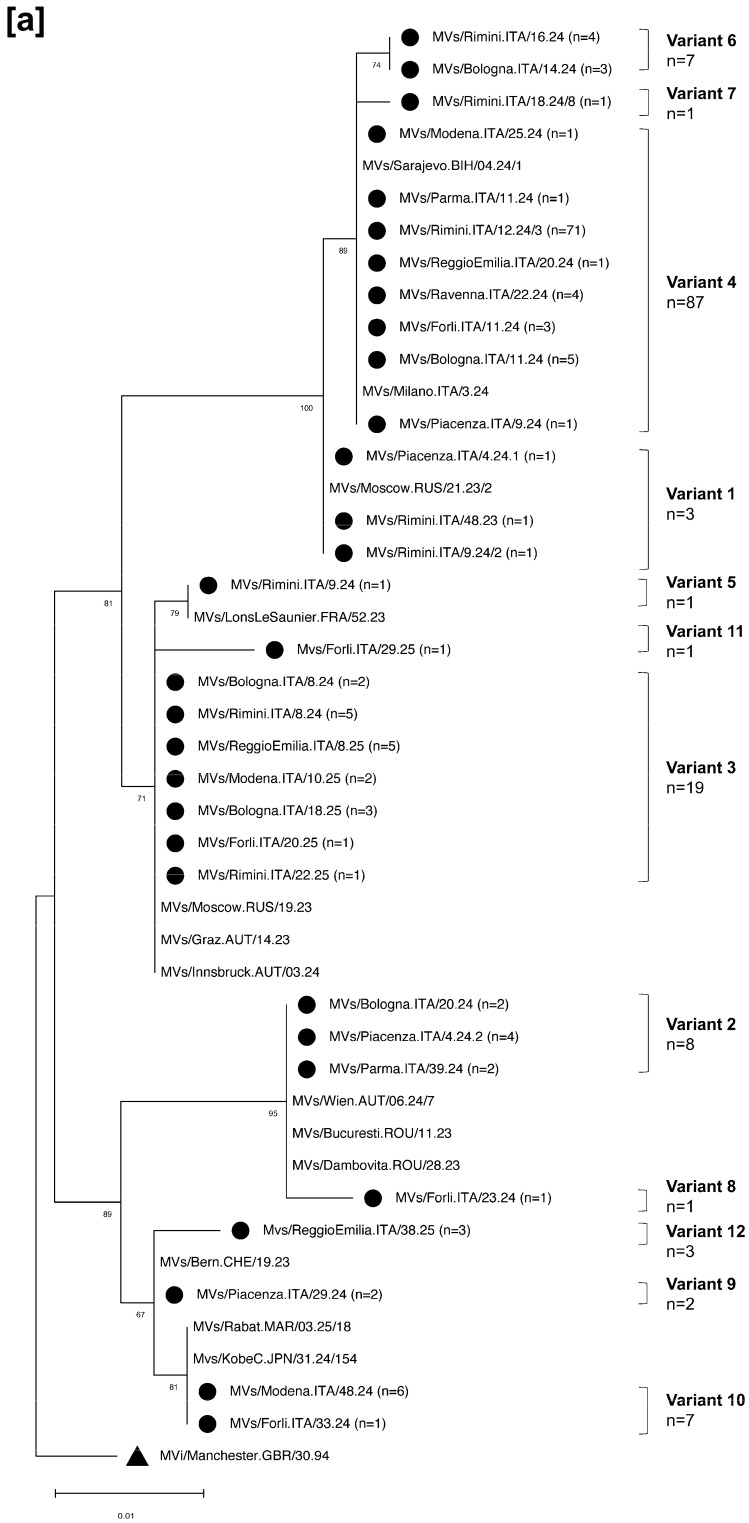

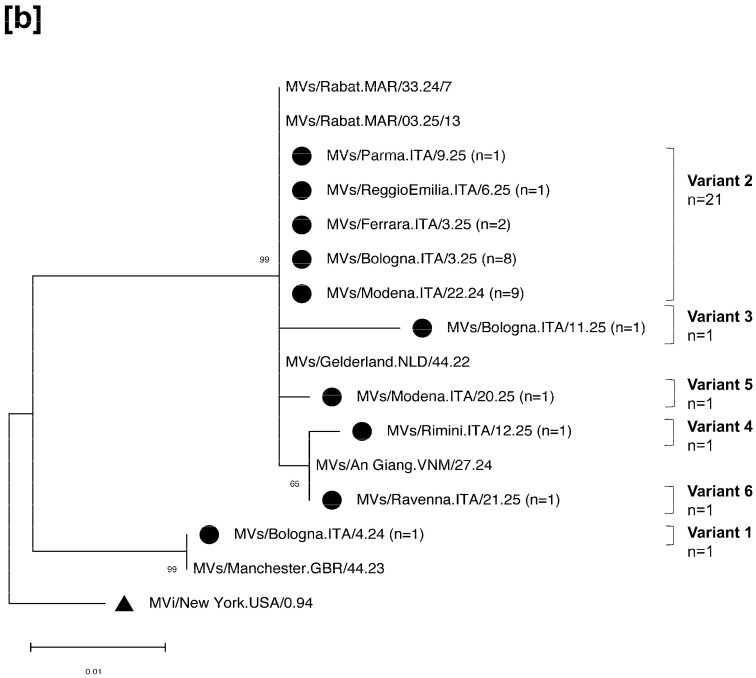
Phylogenetic tree showing MV strains belonging to the genotype D8 (**a**) and B3 (**b**), circulating in ERR. Black circles identify measles strains detected in the area under surveillance. Only one sequence for an identical strain collected in the same year from the same province is reported for each variant (sequences deposited in GenBank with accession numbers: PZ149773, PZ157881–PZ157919, Supplementary Table S3). The evolutionary history was inferred by using the maximum likelihood method and the Kimura 2-parameter model of nucleotide substitutions, and the tree with the highest log-likelihood (−925.20) is shown. Branch support (1000 bootstrap iterations) [36] is provided next to nodes. The initial tree for the heuristic search was selected as the tree with the superior log-likelihood between a neighbour-joining (NJ) tree [37] and a maximum parsimony (MP) tree. The NJ tree was generated using a matrix of pairwise distances computed using the p-distance. The MP tree had the shortest length among 10 MP tree searches, each performed with a randomly generated starting tree. Evolutionary analyses were conducted in MEGA12 using up to seven parallel computing threads [38]. The tree was constructed using D8 and B3 genotype reference sequences (MVi/Manchester.GBR/30.94 and MVi/New York.USA/0.94) as outgroups.

Sequences belonging to genotype D8 clustered into 12 different variants, numbered 1–12 according to the chronological order of detection in ERR (corresponding DSIds are reported in Supplementary Table S2). These strains showed 100% identity with those circulating between 2023 and 2025 in Austria, Bosnia and Herzegovina, France, Italy, Japan, Malaysia, Morocco, the Philippines, Romania, Russia, and Switzerland. Interestingly, the most representative D8 variant in ERR was variant 4 (87/140, 62.1%), which circulated in Italy in 2024 [34] (Figure 5a). The 26 B3 genotype sequences clustered into six different variants, numbered from 1 to 6 according to the chronological order of detection in ERR (Supplementary Table S2). The majority of these sequences (21/26, 80.7%) belonged to variant 2 and were 100% identical to strains circulating in Morocco (Figure 5b). Notably, this variant was first detected in ERR in week 22 of 2024 among indigenous cases and was reintroduced in week 3 of 2025 through imported cases. As described by other authors, this variant characterised the epidemiological scenario of Northern Italy in 2025 [35].

**Figure 6:**
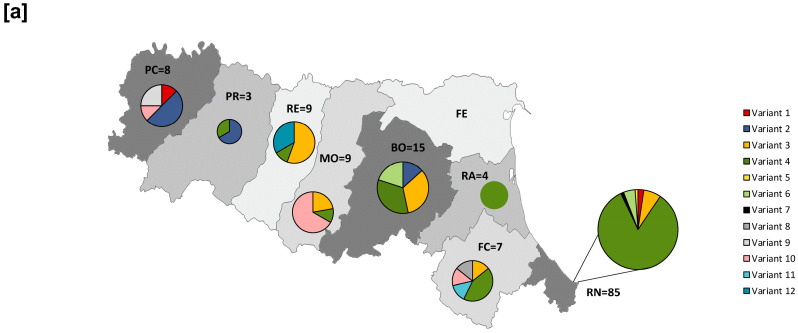

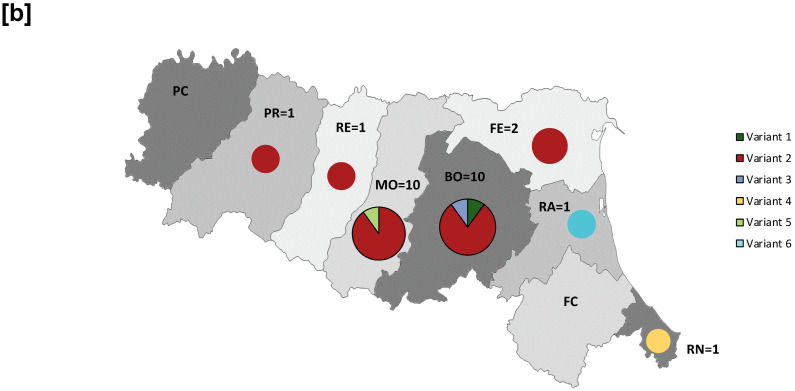
Distribution of genotype D8 (**a**) and B3 (**b**) variants in the ERR. PC: Piacenza; PR: Parma; RE: Reggio-Emilia; MO: Modena; BO: Bologna; FE: Ferrara; RA: Ravenna; FC: Forlì-Cesena; RN: Rimini.

To evaluate the type of transmission (sporadic, outbreak/chain of transmission-related) in association with the source of infection (indigenous, imported, or importation-related) for each D8 and B3 variant, an overview of cases is provided in Table 1. The majority of confirmed cases, which belonged to variant 4, were indigenous and outbreak/chain of transmission-related. In addition, the distribution in ERR of variants belonging to genotype D8 and B3 was shown in Figure 6.

### 3.3. Measles/Rubella Discarded Cases and Differential Diagnosis

According to the laboratory results, 89 of 270 cases (33%) were classified as measles/rubella discarded cases. One of them was vaccinated according to the national immunisation schedule [39] ten days before rash onset and was classified as discarded due to the identification of an MV strain belonging to genotype A (vaccine strain).

**Figure 7:**
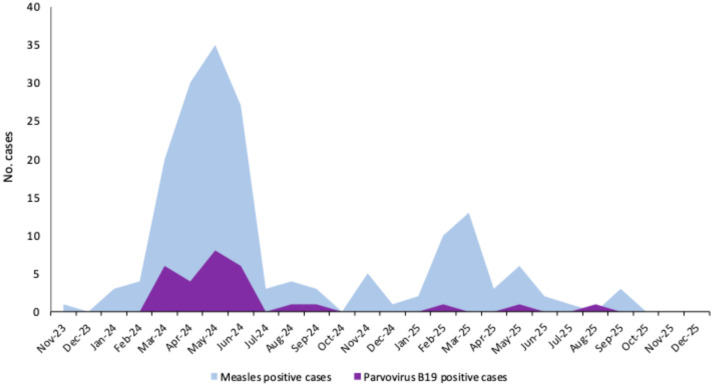
Number of measles and parvovirus B19 positive cases between November 2023 and December 2025.

Among the remaining 88 discarded cases, 77 (87.5%) were tested for B19V-DNA based on serum sample availability, and 37.6% (29/77) were positive. B19V DNA was detected in both children and adults, with a median age of 32 years among infected individuals (range: 5–72 years). Notably, the peak in B19V-positive cases coincided with the peak of confirmed measles cases observed between March and June 2024 (Figure 7). A comparison of clinical symptoms between confirmed measles cases and B19V-positive cases was performed (Supplementary Table S4). In particular, respiratory symptoms were observed in almost all measles cases (161/176, 91.5%), but only in 9 of 29 B19V-positive cases (31.0%). Additionally, other rash-associated viruses were detected: HHV6 (*n* = 1) and EV (*n* = 2); no VZV and DENV positive cases were found. One measles/rubella discarded case tested positive for CHIKV during an autochthonous CHIKV outbreak in ERR. Differential diagnosis identified the etiological agent in 37.5% of measles/rubella discarded cases.

## 4. Discussion

The global resurgence of measles, as reported by the WHO, represents a serious public health issue. In 2023, approximately 10.3 million people were infected with measles worldwide [6]. Particularly concerning is the situation in the European region, which experienced a 30-fold increase in cases and recorded, in 2024, the highest number of cases since 1997 [40]. Multiple factors contributed to this alarming increase, including a decline in vaccination rates, increased international travel, and the removal of social and public health measures following the COVID-19 pandemic [6,7]. In this scenario, Italy was one of the most affected countries, experiencing a marked rise in measles cases throughout 2024 and into early 2025 [10,11,12]. This situation warrants urgent action [7,8,40].

To our knowledge, this is the first study that comprehensively presents the results of both virological and epidemiological surveillance activities—including differential diagnosis data—between November 2023 and December 2025, a period marked by measles re-emergence. This work highlights different crucial aspects that must be addressed to achieve measles elimination.

Firstly, our results showed that 60.5% of suspected measles cases were confirmed through laboratory diagnosis, and of these, 88.1% were detected using both serological and molecular methods. This high confirmation rate reflects the robustness of the integrated surveillance framework. Indeed, the combined use of serological and molecular methods, together with the collection and testing of multiple specimen types (serum, urine, and oral fluid) for each suspected case, increased diagnostic sensitivity, enabled timely case classification, and reduced the risk of misclassification, particularly in atypical presentations or vaccinated individuals. This strategy is essential to interrupt transmission chains and prevent further outbreaks.

Secondly, integrating the molecular characterisation of MV strains with epidemiological investigations provided critical insights into transmission dynamics. Two distinct genotypes, D8 and B3, were observed in ERR during the period under review. Genotype D8 predominated during the 2024 epidemic, with circulating MV strains clustered into twelve distinct variants. In contrast, genotype B3 became increasingly predominant in 2025, with molecular analyses identifying six different variants. This pattern aligns with the circulation observed in other Italian regions [11,35]. Eighty-three percent (146/176) of confirmed cases were classified as indigenous, of which 49.3% (72/146) were sporadic and 50.7% (74/146) related to outbreaks or chains of transmission. The identification of the same D8 variant (variant 4) in the most of indigenous cases suggests ongoing endemic transmission.

In addition, 17% (30/176) of cases were classified as imported, including four importation-related cases, with a travel history to countries such as Morocco and Romania, where major measles outbreaks occurred in 2024–2025 [8,41,42]. Sequencing analysis revealed that 30% of these belonged to a single B3 variant (variant 2). This variant was identical to a B3 variant circulating in Morocco and was responsible for one of the 36 outbreaks that occurred in ERR, reported in epidemiological week 5 of 2025 among passengers on a flight from North Africa to Bologna. These findings support the hypothesis that an importation event followed by local transmission contributes to the rise in measles cases. They also highlight the importance of combining molecular and epidemiological investigations to prevent measles spread and eliminate endemic transmission. Epidemiological investigation is crucial in distinguishing between indigenous and imported cases, providing data on the type of transmission and the source of infection, whereas molecular characterisation mainly supports these findings for outbreak control. Virological surveillance, based on N-450 region sequencing, is affected by low-resolution genomic data. This limit could be overcome by whole-genome sequencing (WGS), which provides accurate and comprehensive information on mutation patterns associated with transmission dynamics. Of note, in a high-quality measles surveillance system, prompt case identification through laboratory diagnosis and immediate contact tracing are key elements in controlling MV transmission. However, variant identification plays a complementary role in tracing transmission chains and should be rapidly performed after laboratory diagnosis. In fact, this is the only way to distinguish between wild-type MV and vaccine strains, especially in symptomatic patients who received a measles-containing vaccine as post-exposure prophylaxis in an outbreak setting. Notably, case classification and molecular characterisation were performed by a Regional Reference Laboratory accredited within a structured network (MoRoNet). This organised collaboration ensures high-quality diagnosis and reliable variant identification, which are essential components of measles elimination strategies.

Our study also highlights that the majority of confirmed cases (90.3%) occurred in unvaccinated individuals, primarily affecting adolescents and young adults (median age: 33 years). These results are in line with national data and European Centre for Disease Prevention and Control (ECDC) reports, which indicate, in some countries, a shift in measles incidence toward older age groups [10,12,35]. In particular, in Italy, France, Poland, and Spain, the proportion of cases aged 30 or older was 52.4%, 28.4%, 34.4%, and 38.5%, respectively. Similar patterns were observed in Germany and the Netherlands [8]. This contrasts with Romania’s child-dominated patient demographic (median age ~5 years), which drove the EU/EEA overall median age downward [9]. These heterogeneous patterns underscore persistent immunity gaps arising from varying historical vaccination strategies and coverage, compounded by inadequate mechanisms for catch-up vaccination to address emerging gaps in older children and adults, as emphasised in the WHO’s Measles and Rubella Strategic Framework 2021–2030 [43]. The recent resurgence of measles is largely attributable to a decline in vaccination coverage, especially during the COVID-19 pandemic (2020–2022), which led to an increase in the number of unvaccinated or under-vaccinated individuals and growing vaccine hesitancy [8,40]. Despite the pandemic, Italy has maintained high vaccination coverage among young children [44]. After the introduction of mandatory measles–mumps–rubella (MMR) vaccination for individuals up to 16 years of age in 2017, national and regional data show that MMR coverage in ERR is above 95% (recommended threshold) at 24 months of age, while it remains below the recommended threshold at 7 years of age [39,44,45]. Altogether, these factors could explain why in our study the current rise in measles cases primarily affected individuals aged 15–39 years, especially in an area of ERR with a high number of vaccine-hesitant communities. In contrast, while many measles cases among healthcare workers were reported at the national level [11,12,34], only 1.7% of cases occurred in healthcare settings in ERR, and none involved HCWs employed at the National Health Service. This result can be attributed to a regional policy requiring HCWs to be vaccinated or to provide documented evidence of previous infection (Regional Resolution No. 351 of 12 March 2018). This underscores the importance of HCWs vaccination, as recommended by the WHO and ECDC [8]. Additionally, among the confirmed cases, complications occurred in 19.3% patients (such as pneumonia and diarrhoea), with hospitalisation required for 68% of individuals. Except for one case, all patients were unvaccinated.

To investigate discarded measles cases, a differential diagnosis including other rash-associated viruses other than RV was performed. Overall, 37.6% of discarded cases tested positive for B19V, indicating an overlapping circulation of B19V with the measles surge in ERR from March to June 2024. This result reflects the ECDC alert on the increase in B19V infections reported in several European countries [17], including other Italian regions [18,19]. Additionally, two discarded cases tested positive for EV: one for HHV-6 and one for CHIKV. This finding underlines the need to integrate arboviral infections into differential diagnosis, especially in the context of travel-related cases or during autochthonous outbreaks. A comparison of clinical manifestations between confirmed measles and B19V-positive cases showed that the two infections shared several overlapping clinical features. However, some symptoms—such as fever and respiratory manifestations—were more frequently observed in confirmed measles cases. In this context, laboratory-based differential diagnosis is crucial to accurately determine the causative agents, especially during periods of co-circulation of several pathogens presenting with similar clinical conditions.

## 5. Conclusions

In conclusion, the study provides valuable insights into key aspects of effective measles surveillance. Although data are limited to a single Italian region, it demonstrates that to eliminate measles transmission, as targeted by the WHO, timely diagnosis, as well as molecular and epidemiological investigations, and accurate case classification are essential. Additionally, it emphasises that close cooperation among different professional figures at both regional and national levels is necessary to strengthen measles and rubella surveillance.

## Figures and Tables

**Table 1:** Overview of variants associated with confirmed D8 and B3 genotype strains identified in ERR between November 2023 and December 2025.

	Variants	No. Cases n = 166	No. Outbreak/Chain of Transmission-Related Cases			No. Sporadic Cases	
			Indigenous n = 69	Imported n = 5	Importation-Related n = 4	Indigenous n = 68	Imported n = 20
D8	Variant 4	87	45	0	0	39	3
	Variant 3	19	7	0	0	8	4
	Variant 2	8	5	0	0	2	1
	Variant 10	7	2	0	0	4	1
	Variant 6	7	5	0	0	2	0
	Variant 1	3	0	0	0	0	3
	Variant 12	3	0	1	1	1	0
	Variant 9	2	0	1	1	0	0
	Variant 5	1	0	0	0	0	1
	Variant 7	1	0	0	0	1	0
	Variant 8	1	0	0	0	0	1
	Variant 11	1	0	0	0	1	0
B3	Variant 2	21	5	3	2	7	4
	Variant 1	1	0	0	0	1	0
	Variant 3	1	0	0	0	0	1
	Variant 4	1	0	0	0	0	1
	Variant 5	1	0	0	0	1	0
	Variant 6	1	0	0	0	1	0

## Data Availability

The original contributions presented in this study are included in the article/Supplementary Material. Further inquiries can be directed to the corresponding author.

## Supplementary Materials

The following supporting information can be downloaded at: https://www.mdpi.com/article/10.3390/diagnostics16071109/s1, Supplementary Table S1: Molecular and/or serological results for the 176 confirmed measles cases; Supplementary Figure S1: Epidemiological investigation of confirmed cases; Supplementary Table S2: Number of D8 and B3 variants with corresponding MeaNS DSId identifier; Supplementary Table S3: GenBank accession numbers of sequences; and Supplementary Table S4: Comparison of clinical symptoms between confirmed measles and parvovirus B19-positive cases.
